# Instructional Design and Its Usability for Branching Model as an Educational Strategy

**DOI:** 10.7759/cureus.39182

**Published:** 2023-05-18

**Authors:** Fernando D Argueta-Muñoz, Hugo E Olvera-Cortés, Cassandra Durán-Cárdenas, Laura Hernández-Gutiérrez, Samuel E Gutierrez-Barreto

**Affiliations:** 1 Medical Education and Simulation, National Autonomous University of Mexico, Mexico City, MEX; 2 Neurosurgery, National Autonomous University of Mexico, Mexico City, MEX

**Keywords:** serious gaming, undergraduate and graduate medical education, delphi method, instructional approach, gamification technique

## Abstract

Introduction: Serious Games (SG) are an educational strategy used in the health professions with positive results in teaching diagnosis and facilitating the application of concepts and knowledge transfer. A type of SG is the branching scenario, which has the potential for a linear story or multiple options to achieve learning goals. There must be evidence for this type of SG's instructional design (InD) and usability. Objective: Propose an InD for the branching scenario and rate its usability.

Materials and methods: We conducted a two-phase study. In the first phase, we drafted an InD based on the literature review, and then, we applied an expert validation process through a modified Delphi technique. With the consent of InD, we built five branching scenarios. In the second phase, we apply an instrument to measure the SG usability of the branching scenarios in a cross-sectional study with 216 undergraduate medical students.

Results: A proposal for an InD for branching scenarios was elaborated. This InD has five dimensions with steps and definitions that help the designer fulfill the requirements for the SG. With the InD, we developed five branching scenarios for undergraduate medical students. Finally, the rates for the usability of the branchings had high scores. The branching SG with multiple options offers different outcomes for the same clinical problem in a single activity.

Discussion: The proposal of a specific InD for branching scenarios considered SG theory and was tested, at least in user usability. The steps proposed include the specificity of the requirements of an SG, such as levels, checkpoints, avatars, and gameplay characteristics, among others, in contrast to the other InD that do not explicitly consider them. One of the limitations of this study is that we applied it only using the H5P software to develop branching scenarios with no other evidence of the performance of the InD in different contexts or platforms.

Conclusions: We propose using an InD to construct branching scenarios. This kind of SG has specific characteristics for its correct operation. Using structured steps in developing SG improves the probability of developing decision-making skills. Using an instrument to assess the usability of at least one dimension of the SG is also recommended to identify opportunity areas.

## Introduction

The paradigm shifts from teaching to learning have placed the student at the center, and this paradigm shift promotes self-assessment, reflection, and learner proactivity in acquiring knowledge. Serious Games (SG) are student-centered learning techniques based on gamification theory. Gamification in education is defined as using game mechanics, dynamics, and frameworks to promote desired behaviors in students using a motivational approach to have a psychological outcome and further behavioral change [1,2]. Gamification supports the learning journey and can be used as a principal guide for education or as a complement to traditional learning [3].

One of the gamification strategies is SG, an educational strategy employed in different areas of knowledge, including medical education [4]. The SG is an educational strategy that aims to acquire knowledge for decision-making rather than entertainment [5,6]. SG recreates situations like those faced by professionals in everyday life with the advantage of not presenting negative consequences [7]. Generally, it allows the participant to analyze their mistakes and correct them to achieve the game's goal. The chance to correct their errors enables the student to increase their ability to take risks, solve problems and make decisions while immersed in a learning scenario in a flexible, personalized environment that simulates specific situations [8].

Medical students who use SG are more than twice as likely to make the correct diagnosis, facilitating the application and transfer of concepts and knowledge [9,10]. One way to use SG is through the branching scenario, which consists of a game where the user advances through a linear or multiple-component story to achieve a clinical goal [11]. The branching model has been utilized in clinical nursing education with promising outcomes for decision-making skills [12]. There are two general ways to develop a branching SG type; linear or multiple branches.

The first, the linear branching type, focuses on following a predefined algorithm in a controlled virtual environment. The main objective of this branching is that the students make decisions to apply and learn new knowledge of only one pathway. The second one is the multiple-ending branching which is more like a real case with more than one pathway. The main objective of this branching is to explore the student's decision-making process. An advantage is that this scenario is similar to an actual situation so it can contain different clinical outcomes according to the decisions taken. Each decision must be analyzed and developed to make the student rethink what they know about the decision options and make the more convenient choice.

Decision-making is a skill that is difficult to develop. This skill is critical in the healthcare environment because it allows health professionals to consider the different circumstances of each patient and make an informed and reasonable decision according to the problem presented. Decision-making is a complex skill to teach and perform because it has cultural, individual, and social elements that could guide the physician's final decision [10]. Using instructional models could contribute to creating educational strategies that adapt to the specific necessities of the target population. Branching scenarios can focus on developing decision-making skills by showing different outcomes of decisions in different or similar clinical situations and contexts.

So far, developing a specific InD to elaborate SG is necessary, especially in branching-type scenarios [13,14]. Some principles have been proposed to design SG for student motivators but do not guide development. Therefore, this study aims to propose an InD for branching scenarios and rate the usability of five branching scenarios.

## Materials and methods

We performed a two-phase study. First, we drafted an InD based on the literature review, and then we applied an expert validation process through the Delphi technique. With the consensus for InD, we built five branching scenarios. In the second phase, we use the instrument elaborated by Bernal and Cols'cols to measure SG usability with the SUS scale in a cross-sectional study [15].

The first phase - Instructional design

Through a literature review, we establish the steps to build an SG. We consider three SG models; the experiential gaming model, the ontology game project, EMERGO, and the recommendations proposed by various authors to construct the steps [16,17]. These models' components include game design, gameplay, usability, and programming. Then we adapted to list steps to use the H5P™ Branching tool embedded in the Moodle™ platform of the School of Medicine (FacMed) of the National Autonomous University of Mexico (UNAM) in 2022.

The steps for branching scenarios were validated with the Delphi method [18]. The participants in the validation process were selected with the following criteria: a) at least five years of experience in medical education, b) at least five years using clinical reasoning educational strategies, and c) having built or used SG. The instrument has a Likert scale from 1 to 5, where one means total disagreement and five means complete agreement; it also has a section for opinions and suggestions. The instrument was sent through Google Forms™ to look for consensus.

Using the InD concept, we constructed SG based on the learning outcomes of undergraduate medical education with free student access. Following Killam et al.'s, we program the SG using the H5P™ interface recommendations [11]. By reaching a consensus, experts assured SG content validity in the field of knowledge, and the authors made the programming of this article.

The second phase - Evaluation of the SG

Fourth-year students at the school of medicine enrolled in 2022 at the FacMed of UNAM participated in the second phase. The sample size was a probabilistic sample calculated for 89 students with the absolute difference criterion for a known population of 1,122 students, a statistical power of 90%, and a confidence level of 95%. The instrument consists of a Likert scale (1 in total disagreement and 5 in complete agreement) of thirty questions that consider the following dimensions: the objective of the game, visualization of the story, visualization of the game world, visualization of characters, gamification techniques, gameplay, interface, and multimedia (SUS scale); additionally, it has two open questions to know the opinion of the participant [15]. The open questions were analyzed with thematic analysis. The instrument was embedded in the Moodle virtual academic classroom through GoogleForms™. The response was collected after using the SG. The SG was presented through the Moodle platform, where the students could play the game any time they wanted for sixteen weeks.

The descriptive analysis of the results was performed with JASP version 0.17.1. All participants were informed of the study's objective at the beginning of the study, and written consent was sought for all participants. The Ethics Committee of the University agreed that we could proceed as a quality assurance project and provided the appropriate permits to carry out in alignment with the declaration of Helsinki.

## Results

As a result of the methodology previously exposed, we presented the findings of the two-phase study. This section will be divided to disclose the results. In Phase 1, we describe the consensus process involving the Delphi technique and the design of the SG branching. In Phase 2, we describe the evaluation process of the utility of the game with Bernal's and Cols' instruments.

First phase - Instructional design

**Figure 1:**
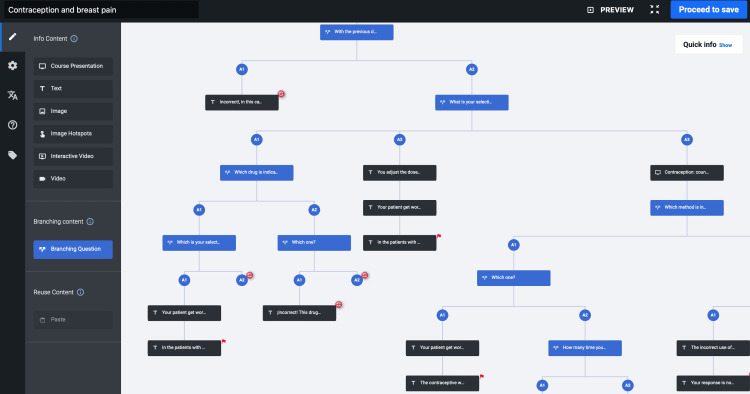
Multi-component branching model This is a representative image from the user screen for the H5P tool. The blue boxes are decision questions and the black ones are only multimedia content. The flags on the boxes represent one of the ends in the branch scenario and the return symbol is an interactive media that can jump between the blue boxes. Finally, the circles are points of reference during the construction of the scenario.

In the first phase, we elaborate on an InD to use in developing SG. To make this possible, we constructed a draft for InD based on the literature review with the following dimensions: analysis, structure, construction, application, and evaluation (Table 1). Each dimension had one or more steps to construct the SG. These dimensions and steps were consistent with the SG design. The consensus participants were eight persons, with a mean of nine years of medical education, six years of experience using clinical reasoning educational strategies, and at least three built SG. The Delphi technique showed that 14 out of the 22 items were sufficient, clear, coherent, and relevant for each dimension. The comments redefined the following dimensions about the specificity of the definitions of the InD; target population, objectives, game features, feedback system, and the knowledge and skills developed. In the second round, the eight items reach a consensus without changes. Finally, we construct the four dimensions of InD with 21 steps. Each step had a series of specifications to design an SG. With the InD consent (Table 2), we developed five serious games, two with multiple branch formats and three with a linear structure using the H5P platform. Figure 1 shows an example of a multiple-branch scenario elaborated with the H5P interface. A clinical expert committee assured the validity of the content of each SG.

**Table 1:** Principal components for the branching model of instructional design.

Analysis	Structure	Construction	Application	Evaluation
Target population	History	Instructions for the target population	Show the game to the target population	Perception of the quality of the game
Objectives	How to play	Construction of the history		Knowledge and skills developed
Game features	Checkpoints and level/story conclusion	Building decision-making components		
Programming system		Construction of feedback components		
Feedback system		Verify functionality		
Cost		Expert review of the final product		
Scope				
Update system				
Interaction between students-tutors-external media				

**Table 2:** Model for the construction of branching scenarios. *SG: serious games

Stage	Step	Description
Analysis	Target Population	Define the population to which SG is directed and recognize its characteristics (academic degree, use of technology, information management).
	Objectives	Define the general and specific objectives. Define learning outcomes.
	Game Features	Specify whether the game is a digital, physical, or hybrid object. Establish characters or avatars and the environment in which it takes place. Determine the usability; how easy it is to use in an intra- or extra-class. Consider how easy it will be to access the game. Test the integration and the interactions of the game elements. Define the website or physical site where the SG will be stored and distributed. Set whether the game is multiplayer or single-player. Set the number of levels and methods to access them.
	Programming system (if required)	Consider the programming time, personal, and costs of the SG. Take into account having trained personnel for this purpose.
	Feedback system	Create a system of rewards and penalties for the decisions made by the player. Consider whether points, objects, feedback, among others, will be used.
	Costs	Establish the budget for development, the necessary equipment, the required licenses, the personnel, etc.
	Scope	Consider whether another population can use serious play in other environments, contexts, and similar situations.
	Update system	Establish the protocol and temporality in which the SG will be updated, according to the objectives achieved or the academic program.
	Interactions between students-tutors-external media	Define the relationship between the users of the SG. Communication between peers, superiors, or cognitive aids to advance within the story.
Structure	How to play	Design the rules and the way components of the game interact.
	History	Establish an algorithm that marks the objectives and learning outcomes within the game. Either by a linear or branched algorithm. Define minimum elements for the achievement of the objectives.
	Checkpoints and level/story conclusion	Set whether it is necessary to have save, pause or reset points in the story or level; according to the decision made.
Construction	Instructions for the target population	Write clear and precise rules for how components, characters, players, and environment interact during SG development. The system of rewards and penalties and save points will have to be specified.
	Construction of history	The story’s plot must be related to the proposed thematic objectives and must be developed so that the student can identify the problem that arises.
	Building decision-making components	Consider that the decision-making points are clear, easily identifiable, and attached to the game’s objective.
	Construction of feedback components	Adhere to objective, clear, timely, concise feedback attached to the most recent literature encourages self-regulated learning.
	Verify functionality	Use each of the components of the game and make sure it works as expected.
	Expert review of the final product	Experts in the field will test the SG before being released to the public to verify its coherence and relevance at all stages of development and verify its veracity and realism.
Application	Show the game to the target population	Consider the use of a beta version. Determine how the target population will use the game.
Evaluation	Perception of quality of the game	Evaluate serious play with an instrument that measures usage satisfaction, gameplay, learning perception, and SG design.
	Knowledge and skills developed	Assess whether the target population acquired the knowledge and skills intended to be achieved with serious play.

Phase 2 - Usability

The target students evaluated the SG's usability 219 responses were obtained. The mean age of the participants was 22, and 66% of the students were female. The residency place of the participants was 95% in México City. Three were eliminated by not accepting informed consent. The results by dimension and the instruments, in general, are in Table 3. The multimedia dimension (D8) was rated best with a low standard deviation (SD). The gameplay (D6) has a lower mean score (4.0) and a higher SD (1.0). The results of the whole instrument had a mean of 4.3 with a 0.7 SD. According to the SUS scale, this is interpreted as 86% or excellent usability [19]. The results presented were the sum of all branching scenario evaluations. The instrument comments were classified into two large groups (advantages and disadvantages) and analyzed thematically by dimension (Table 4). The comments for advantages were the group's outstanding dimensions: decision-making, clinical reasoning, and immediate feedback. The disadvantages were in the dimensions of media playback and cognitive load.

**Table 3:** Descriptive analysis of the dimensions and total of the instrument D1: objective of the game, D2: visualization of the story, D3: visualization of the game world, D4: visualization of characters, D5: gamification techniques, D6: gameplay, D7: interface, and D9: multimedia.

	D1	D2	D3	D4	D5	D6	D7	D8	Total
Mean* (1-5)	4.2	4.2	4.3	4.2	4.1	4.0	4.3	4.4	4.3
SD	0.98	0.92	0.80	0.82	0.96	1.0	0.84	0.80	0.70
Median	5	4	4	4	4	5	5	5	4

**Table 4:** Advantages and disadvantages of using serious games

	Dimension	Comment
Advantages	Decision making	“They were excellent; they made me think twice about my choices.”
	Clinical reasoning	“I liked the interaction presented and the way to promote clinical reasoning.”
	Immediate feedback	“They are outstanding, as you do them, they give you excellent feedback, whether your answer is correct or not, and they justify the possible scenarios that result in each case.”
Disadvantages:	Media playback	“Sometimes, the platform did not load the activities correctly, so it had to be done again. Despite changing the browser, the problem remained. The platform and exercises were friendly in access from there on out.”
	Cognitive load	“They were good, but sometimes I did not like them because they would return you to the first questions, which became tedious, coupled with the failures of the classroom, they made the activities tedious and very long.”

## Discussion

The InD shows specific steps for constructing a branching type of SG in health sciences and evaluating how the final users perceive its usability. A specific InD helps construct a branching type SG systematically, which could contribute to teaching decision-making skills more effectively. In this study, we obtained evidence from the comments made by the students that mentioned the facility of reshaping the decision-making process through the branching scenario. Using a validated InD for branching scenarios improves the usability and probability of success in teaching complex skills such as decision-making.

The InD developed only considered the construct evidence from usability and expert consensus. Otherwise, instructional models such as ADDIE [20] and ASSURE [21] focus on constructing various educational objects in a virtual or physical context. These models have advantages such as simplicity and wide use in designing educational objects. The steps proposed include the specificity of the requirements of an SG, such as levels, checkpoints, avatars, gameplay characteristics, etc., in contrast to the other InD that do not explicitly consider them. This InD can be helpful to branching scenario SG designers due to the reflective steps on this specific SG type.

Educators consider the performance of an SG in different ways, such as knowledge acquired, satisfaction, outcomes, and decision-making [22]. In this case, we considered the SG in terms of product usability. Knowing the game's usability can contribute to user engagement, and making an SG with an InD that has proven to achieve this goal might make a better transfer from the SG to real life. SG has proven helpful in transferring clinical reasoning to patients like those presented virtually [9]. Although usability is one way to assess SG, we obtained promising results that contribute to the content validity of the InD for branching type SG. The transfer of clinical reasoning and skills has to be assessed in further studies.

The usability results of the linear and multiple branching scenarios had no significant differences in the usability instrument. Although, the branching SG with multiple branches offers different outcomes for the same clinical problem in a single activity. This SG could help the students develop clinical reasoning and decision-making for diagnosing and treating numerous clinical entities. Applying the knowledge acquired with SG might improve patient safety [23]. Branching type SG might improve decision-making and clinical reasoning in essential aspects such as treatment and diagnosis [24,25].

By one of its definitions, InD helps create better systems and learning objects that can make learning experiences that develop and enhance skills and knowledge [26]. InD help make branching-type SG offer a different learning experience that can improve the outcomes when treating a patient. One of the limitations of this study is that we applied it only in H5P branching scenarios with no other evidence of the performance of the InD in different contexts or platforms. Further studies are needed to assess the competency transference of the SG to actual performance and the use of InD and decision-making skills.

## Conclusions

The proposed InD has five stages, and the final users evaluated the branching scenarios with good usability. We recommend using an InD for any SG; the proposal for an InD has specific characteristics for its correct operation that permit general use. Despite this, We suggest using the proposal InD model and considering the best suits the students' and users' needs to achieve an optimal SG to fulfill their objectives. Also, we recommend the usability test to measure the branching scenario in a specific context.
